# New Approaches to Target T-ALL

**DOI:** 10.3389/fonc.2014.00170

**Published:** 2014-07-08

**Authors:** Giovanni Roti, Kimberly Stegmaier

**Affiliations:** ^1^Department of Pediatric Oncology, Dana-Farber Cancer Institute, Boston, MA, USA; ^2^Division of Hematology/Oncology, Boston Children’s Hospital, Boston, MA, USA; ^3^Hematology and Bone Marrow Transplantation Unit, University of Perugia, Perugia, Italy; ^4^Broad Institute of Harvard and Massachusetts Institute of Technology, Cambridge, MA, USA

**Keywords:** T-cell acute lymphoblastic leukemia, NOTCH1, cyclins, cyclin-dependent kinases, PI3K/AKT/mTOR, PI3K pathway inhibitors, BRD4, bromodomain inhibitors

## Abstract

Acute lymphoblastic leukemia is the most common malignancy in children. Although it is now curable in 80–90% of cases, patients with T-cell acute lymphoblastic leukemia (T-ALL) experience a higher frequency of induction failure and early relapse. Despite aggressive treatment approaches, including transplantation and new salvage regimens, most children with relapsed T-ALL will not be cured. As such, we are in need of new targeted therapies for the disease. Recent advances in the molecular characterization of T-ALL have uncovered a number of new therapeutic targets. This review will summarize recent advancements in the study of inhibiting the NOTCH1, PI3K–AKT, and Cyclin D3:CDK4/6 pathways as therapeutic strategies for T-ALL. We will focus on pre-clinical studies supporting the testing of small-molecule inhibitors targeting these proteins and the rationale of combination therapies. Moreover, epigenetic approaches to modulate T-ALL are rapidly emerging. Here, we will discuss the data supporting the role of bromodomain and extra-terminal bromodomain inhibitors in human T-ALL.

While cure rates for children with acute lymphoblastic leukemia (ALL) have dramatically improved over the last several decades, ALL still remains a leading cause of cancer-related death in children. For adults with ALL, progress has been rather modest. One high-risk ALL subtype, T-cell acute lymphoblastic leukemia (T-ALL), accounts for 10–15% of pediatric and 25% of adult ALL cases. Although treatment of T-ALL has improved, early relapse is common and is almost invariably associated with poor prognosis. Furthermore, a major challenge remains the lifelong morbidity suffered by patients treated with current chemotherapy regimens. We are in need of more effective and selective treatment strategies. In this review, we will focus on emerging druggable opportunities in T-ALL: NOTCH1, BRD4/MYC, Cyclin D3:CDK4/6, and the PI3K pathway.

## Notch Pathway Mutations

Notch signaling is a critical driver of T-cell differentiation (1), specifically the commitment of lymphoid precursors to the T-cell fate, as well as to subsequent thymocyte development (2–5). Thus, it is not a surprise that aberrancies in Notch signaling are one of the major oncogenic events in T-ALL. Gain-of-function mutations in *NOTCH1* are the most common genetic abnormalities reported in T-ALL. Activating mutations of *NOTCH1* are present in 55–60% of T-ALL cases (6). Chromosomal rearrangements involving *NOTCH1 t*(7;9)(q34;q34.3) have been characterized in human T-ALL and lymphoma (7, 8), and *NOTCH1* mutations have also been reported in 5.3–20% of chronic lymphocytic leukemia (CLL) (9, 10). Mutations that inactivate the Notch pathway have been described in a number of human cancers, such as chronic myelomonocytic leukemia (CMML) (11) and squamous cell malignancies involving the skin, head, and neck (12, 13), indicating that Notch signaling can be either oncogenic or tumor suppressive depending on the cellular context. *NOTCH1* encodes for a transmembrane receptor activated through a series of proteolytic cleavage events. In normal mammalian signaling, canonical NOTCH1 pathway activation relies on ligand-induced (Delta-like 1, 3, 4 or Jagged/Serrate 1 or 2) cleavage of the receptor that results in release of the intracellular domain of Notch (ICN). This process is mediated by γ-secretase, a multi-subunit protease complex that cleaves single-pass type I integral membrane proteins at residues within the transmembrane domain. ICN1 then translocates to the nucleus, associates with other proteins as a member of a transcription factor complex and initiates highly diverse transcriptional programs that govern an array of cellular functions (1).

Notch receptors have a modular domain organization. The ectodomains of Notch receptors consist of a series of N-terminal epidermal growth factor (EGF)-like repeats, responsible for ligand binding, followed by three LIN-12/Notch repeat (LNR) modules that prevent receptor activation. Next, the heterodimerization domain (HD) links the extracellular tail to ICN1, the domain involved in transcriptional regulation. ICN1 consists of a RAM domain, seven ankyrin (ANK) repeats flanked by two nuclear localization signals (NLS), a transactivation domain (TAD), and a PEST region that participates in protein degradation. The majority of leukemogenic mutations are located in either the HD or PEST domains (6). Notch activation through class I HD mutations occurs by single amino acid substitutions or in-frame insertions or deletions that reduce the stability of the LNR–HD complex and generate a constitutively active, ligand-independent Notch protein (6, 14). Class II HD mutations are longer insertions located at the distal part of the HD domain that expose the proteolytic cleavage site (S2) to the activity of the extracellular ADAM metalloprotease causing high levels of ligand-independent activation of NOTCH1 (6). A third class of mutation, juxtamembrane expansion mutants (JME), are internal tandem duplications in the 3^′^ end of intron 27 and/or the proximal region of exon 28, which result in high level of activation due to the increased separation of the HD–LNR repeat complex from the membrane (15). In contrast, *NOTCH1* PEST mutations delete the C-terminal part of the receptor impairing the degradation of activated NOTCH1. Similarly, mutations in *FBXW7*, a gene that encodes an ubiquitin ligase, mimic the effect of *NOTCH1* PEST deletions thus increasing the stability of ICN1. Several studies strongly support the development of NOTCH1 inhibitors for targeted cancer therapy, particularly for T-ALL, where recurrent *NOTCH1* mutations are common and cancer dependency has been well established (16–21). For example, several reports have shown that transgenic expression of ICN1 leads to the rapid development of aggressive T-cell leukemia/lymphomas (7, 20). Furthermore, Demarest and colleagues demonstrated that c-Myc expression cannot fully rescue a T-ALL tumor when *Notch1* expression is extinguished in a transgenic mouse model indicating that tumor maintenance is dependent on oncogenic Notch signaling (21).

**Figure 1:**
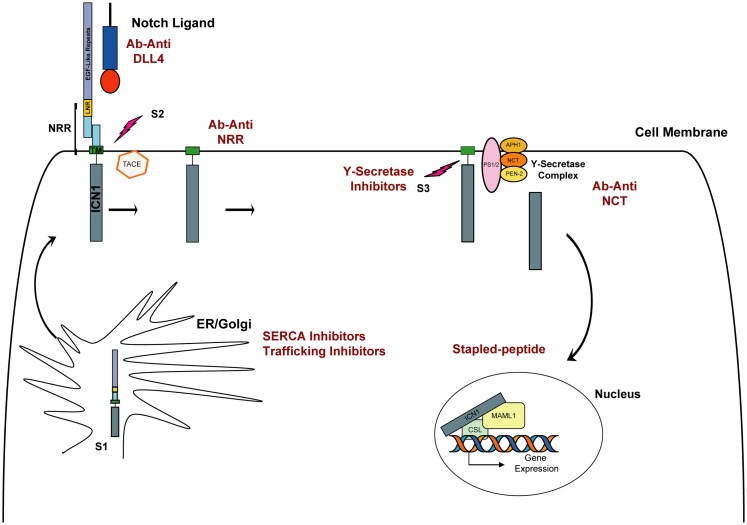
**Schematic representation of NOTCH1 signaling**. The Notch receptor is cleaved by furin (S1 cleavage) in the Golgi and then matures to the cell membrane. In physiological conditions, the binding of Delta or Jagged ligand to the Notch receptor initiates two consecutive proteolytic cleavage events; the first is mediated by the ADAM protease TACE and occurs on the extracellular side of Notch near the transmembrane domain (S2). The second cleavage (S3) occurs within the transmembrane domain and is mediated by the activity of γ-secretase, a complex composed of four proteins: presenilin (PS1/2), nicastrin (NCT), APH-1, and PEN-2. Intracellular NOTCH1 (ICN1) is released and translocates to the nucleus. In the nucleus, ICN1 binds to the CSL transcription factor, converting it from a transcriptional repressor into a transcriptional activator while recruiting the coactivator MALM1. In red are indicated approaches to inhibit Notch signaling.

Given NOTCH1’s important role in the pathogenesis of T-ALL, and its activation through a series of proteolytic cleavage events, it is a propitious target for drug development (Figure 1). The first and most comprehensively studied approach to targeting NOTCH1 is the inhibition of the γ-secretase complex. γ-Secretase inhibitors (GSIs) were originally identified in cell-based drug screens for inhibitors of β amyloid production, considered a causative event in the development of Alzheimer disease (AD). Because the γ-secretase complex is a chemically tractable target, several potent, orally available, brain penetrant small molecules have been developed and tested in pre-clinical studies and in humans with AD and cancer. Despite promising pre-clinical studies with the GSI MK-0752 in *NOTCH1*-mutated T-ALL cell lines (16, 22), the first phase I clinical trial testing this molecule in patients with T-ALL showed limited antitumor activity, and the continuous dosing regimen was associated with severe gastro-intestinal (GI) toxicity (23). Subsequently, it was demonstrated that GSI inhibition leads to a decrement in normal NOTCH1 and NOTCH2 signaling in the GI tract causing alteration of the proliferative compartment and accumulation of mucus-secreting goblet cells in the gut epithelium, thus explaining the severe diarrhea in treated patients (24, 25). Although these side effects preclude the continuous administration of single agent GSIs, they can be attenuated with intermittent drug dosing. Alternatively, pre-clinical studies demonstrated that the combination of a GSI and dexamethasone dramatically attenuated GI toxicity and showed enhanced anti-leukemia properties in T-ALL models (24, 26, 27).

An alternative approach to targeting Notch has focused on the development of antibody-based therapy directed against specific components of Notch receptors and their ligands. Because the Notch-Delta-Like 4 (DLL4) axis lies downstream of initiating signals induced by vascular-endothelial growth factor (VEGF), its inhibition has been regarded as a promising new targeted therapy in tumor angiogenesis (28). To inhibit the NOTCH1–DLL4 receptor–ligand interaction different approaches have been used, including the development of monoclonal antibodies (29), decoy ligands (30), and decoy receptor molecules (31, 32). Preliminary studies showed tumor regression using this approach (29, 33), although prolonged treatment has been associated with the development of vascular-endothelial cell-based tumors and liver toxicity in mice, necessitating an intermittent dosing schedule (34–36). More relevant for a T-ALL application is the development of antibodies inhibiting Notch signaling independently from its interaction with ligands. Inhibitory antibodies against the NOTCH1 (35, 37) and NOTCH2 (37) negative regulatory region (NRR) have been reported. By targeting the NRR region, which spans from the LNR to the HD domain, both groups identified a mechanism to lock Notch signaling in the off state. Both demonstrated that anti-NRR antibodies inhibit growth of NOTCH1-driven T-ALL cell lines. Furthermore, Wu and colleagues demonstrated that anti-NRR1 can dramatically decrease growth in pre-clinical xenograft models (37). Interestingly targeting NRR1 or NRR2 had no effect on weight in treated mice, a remarkable contrast with the nearly 20% weight loss caused by inhibiting both receptors simultaneously, a known undesired effect previously observed in animal models and patients treated with GSIs (37). These first studies on selective inhibition of NOTCH1 and NOTCH2 showed that individual dosing with isoform-specific antibodies generated few (NRR1) or no (NRR2) changes to intestinal morphology and so suggest that selective inhibition of Notch receptors is feasible and effective in T-ALL models and leads to diminished gut toxicity.

An additional example relies on the development of antibodies raised against components of the γ-secretase complex, such as nicastrin. A5226A, a monoclonal antibody against the extracellular domain of nicastrin, inhibits the γ-secretase activity by competing with the substrate binding *in vitro*. Only a moderate decrement of ICN1 was observed upon A5226A treatment in DND41 cells *in vitro*, but a significant reduction in tumor volume for DND41 xenotransplants was observed (38).

While pre-clinical studies strongly support the development of new Notch inhibitors in human cancers, the discovery of alternative Notch pathway antagonists with a different mechanism of action from GSIs or Notch-directed antibodies represents a challenge. Historically, transcription factors have been considered among the most chemically intractable of protein targets because they mediate their action largely through protein–protein and protein–DNA interactions rather than enzymatic activities and because designing high-throughput screening assays to measure these interactions is difficult. One potential approach to directly target the Notch complex might use a stapled peptide technology (39). A second is based on the premise that one can target a transcription factor abnormality by identifying compounds that successfully target its deranged transcriptional program (40).

Our laboratory applied Gene Expression-based High-throughput Screening (GE-HTS) (41), a screening approach that leverages the assessment of gene expression signatures as surrogates for different biological states. A 28 gene expression signature for the NOTCH1 activated state was defined and adapted to the GE-HTS assay, which uses ligation-mediated amplification (LMA) and a Luminex bead-based detection system. Three thousand eight hundred one compounds were screened and results of the chemical screen integrated with those of a complementary cDNA library screen for factors that enhance the signaling activity of the NOTCH1 mutant L1601PΔP. The P-type ATPase SERCAs emerged at the nexus of these two complementary screens as a potential therapeutic target in NOTCH1-associated T-ALL. Briefly, the SERCA inhibitor thapsigargin down-regulated the expression of the NOTCH1 target genes, and similar to GSIs, induced a G0/G1 arrest and a decrease in cell size. We demonstrated that thapsigargin inhibits Notch signaling by altering its normal maturation. Treatment with low nanomolar concentrations of thapsigargin led to a marked decrement in transmembrane and activated NOTCH1 while unprocessed, full-length NOTCH1 receptors accumulated in the endoplasmic reticulum (ER)/Golgi compartment. This result was supported by the prior observation that *Ca-P60A* (SERCA homolog) deficiency leads to a Notch maturation defect in *Drosophila* (42). We also confirmed that SERCA inhibition induces on target anti T-ALL activity *in vitro* and in T-ALL xenograft models *in vivo*. Moreover, T-ALL cell lines expressing leukemogenic *NOTCH1* HD domain mutations were more sensitive to the SERCA inhibitor thapsigargin than normal receptors, supporting the possibility of a therapeutic window for compounds of this class (40).

Subsequent studies confirmed that targeting the Notch secretory pathway may represent an alternative mechanism to inhibit Notch signaling. Kramer and colleagues (43) developed a high-content screening assay to identify new regulatory proteins involved in Notch trafficking and processing in human cells. This assay relied on the generation of constitutively active, ligand-independent, NotchΔE-eGF stable cell lines in which nuclear eGFP staining was the surrogate measure of Notch activation. The authors identified four γ-secretase modulators and the dihydropyridine FLI-06. They demonstrated that FLI-06 inhibits Notch trafficking early in the protein secretary pathway (43). Although the precise protein target of FLI-06 is yet to be determined, this finding is particularly important because new candidate targets/molecules regulating Notch trafficking are rapidly emerging in studies of Notch in other model organisms (44).

## BRD4/MYC

The bromodomain (BRD) is a conserved protein motif of ~110 amino acids that recognizes and binds ε-*N*-acetylated lysine residues in histone and non-histone proteins. Through this interaction, bromodomain-containing proteins facilitate the anchoring of nuclear macromolecular complexes to specific acetylated nucleosome sites on chromatin and control several biological processes including DNA replication, DNA damage repair, chromatin remodeling, and transcription regulation (45). The bromodomain and extra-terminal (BET) family of proteins, defined by tandem BET domain, include BRD2, BRD3, BRD4, and BRDT. BET proteins play a key role in many cellular processes, including gene expression, mitosis control, and viral–host interaction and more recently have emerged as potential therapeutic targets in cancer (46).

**Figure 2:**
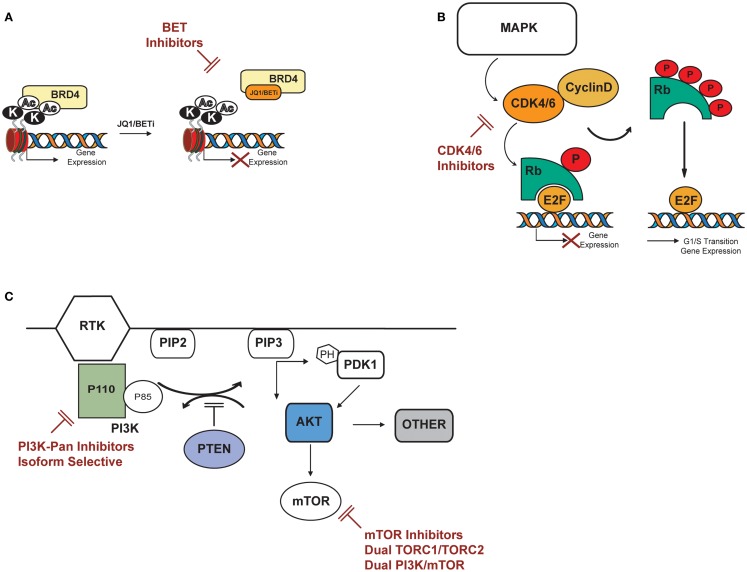
**Potential targets in T-ALL: PI3K/AKT/mTOR, cyclin D3:CDK4/6, BRD4/MYC**. **(A)** BET bromodomain inhibitors displace BET bromodomain, such as BRD4, from chromatin by competitively binding to the acetyl lysine recognition pocket and lead to the repression of BRD4 transcriptional targets. **(B)** CDK4/6 inhibitors specifically inhibit CDK4 and 6, thereby inhibiting retinoblastoma (Rb) protein phosphorylation in early G1. Inhibition of Rb phosphorylation prevents CDK-mediated G1-S-phase transition, thereby arresting the cell cycle in the G1 phase, suppressing DNA synthesis, and inhibiting cancer cell growth. **(C)** PI3K catalyzes phosphorylation of the D3 position on phosphoinositide to generate the second messenger phosphatidylinositol-3,4-5 trisphosphate (PIP3) from phosphatidylinositol-3,4 bisphosphate (PIP2). PIP3 binds to the pleckstrin homology (PH) domains of the 3^′^ phosphoinositide-dependant kinase (PDK-1) and AKT causing both proteins to translocate to the cell membrane where they are subsequently activated. AKT activation stimulates metabolism, cell-cycle progression, survival and migration through phosphorylation of many physiological substrates, including mTOR. The tumor suppressor PTEN inhibits PI3K signaling by the dephosphorylation of PIP3. AKT can activate mTOR directly by phosphorylation at S2448 or indirectly by inhibition of the tuberous sclerosis complex TSC2. Potential inhibitors of the above described pathway are indicated in red.

The last year has seen the rapid development of multiple small-molecule inhibitors of BET bromodomains by both academic groups and pharmaceutical companies, with JQ1 among the first in class. JQ1 is a novel thieno-triazolo-1,4-diazepine that binds selectively and with high affinity to the acetyl lysine pocket of the conserved BET domains of the BRD protein family (Figure 2A). Its activity was first described in NUT midline carcinoma, a rare, aggressive epithelial cancer genetically defined by a chromosomal translocation of *BRD4* with *NUT* (47), where JQ1 inhibition of BRD4 induced squamous cell differentiation and tumor regression in a tumor primagraft model (48). Evidence of BRD4 dependency in human malignancies was later extended to acute myeloid leukemia (AML). In screening a short hairpin RNA (shRNA) library targeting chromatin regulators in an MLL-AF9 model of AML, BRD4 was found to be critical for disease maintenance, and inhibition with JQ1 ablated the expression and function of *MYC* itself (49). This compound class has been reported to be active in a number of diseases dependent on either MYC or MYCN, including neuroblastoma, medulloblastoma, multiple myeloma, and non-Hodgkin Lymphoma (NHL), to name a few (50–54).

In T-ALL, multiple lines of evidence now suggest a role for targeting MYC with BET bromodomain inhibitors. First, NOTCH1-mediated activation of MYC is important to the maintenance of leukemic growth (55). Second, two recent studies support the role for MYC in T-ALL leukemia initiation and demonstrate anti-leukemic activity with BET bromodomain inhibitors, including JQ1. In a study by King et al., dependence on MYC for leukemia-initiating cell (LIC) activity was demonstrated using a mouse model of the Fbxw7^R468C^ substitution, an event commonly observed in human T-ALL leading to the stabilization of NOTCH and MYC proteins (56). These investigators next explored the inhibition of BRD4 as a therapeutic strategy for downregulating MYC in T-ALL. They demonstrated that knockdown of *Brd4* and *Myc* inhibited growth in Notch1-positive mouse T-ALL cell lines, and JQ1 treatment showed a significant dose-dependent decrease in cellular growth along with reduction in MYC protein expression in a panel of T-ALL cell lines and in primary human T-ALL samples tested *in vitro*. This decrease in cellular growth could be partially rescued with overexpression of a *MYC* transgene. Human *FBXW7* mutant T-ALL cell lines, that are typically GSI resistant, were also sensitive to treatment with JQ1. Furthermore, a BET bromodomain inhibitor was demonstrated to have efficacy *in vivo* in both Fbxw7^+/+^ and Fbxw7^R468C/+^ mouse models of T-ALL (56).

An independent study by Roderick et al. also demonstrated the importance of MYC in the LIC in T-ALL. In this study, c-Myc abrogation depleted leukemia LICs and prolonged survival in a *Notch1* mutant Tal1/Lmo2 T-ALL mouse model (57). Pharmacological inhibition with JQ1 of Tal1/Lmo2 T-ALL murine cell lines demonstrated that Brd4 inhibition induced an initial cell-cycle arrest followed by apoptosis, in contrast to GSI, which predominantly induced cell-cycle arrest with minimal apoptosis. These effects were partially rescued by the overexpression of an exogenous murine *c-Myc* construct supporting that c-Myc is a relevant target of Brd4 in mouse T-ALL. In order to test whether JQ1 interferes with leukemia initiation and reduces LICs, Tal1/Lmo2 mouse T-ALLs were transplanted into syngeneic recipients and vehicle or JQ1 administered for 3 weeks. JQ1 significantly increased overall survival and depleted LICs, further supporting the critical role of c-Myc in mouse LIC maintenance. Further testing of JQ1 confirmed that BRD4 inhibition impaired the growth and induced apoptosis in human T-ALL cell lines. In 8 out of 10 primary T-ALL pediatric samples JQ1 inhibited cell growth and reduced MYC levels. JQ1 was active even in cases of refractory or relapsed T-ALL. Additional data generated in high-risk primary T-ALL short-term culture assays demonstrated that JQ1 induced apoptosis and the expression of the pro-apoptotic factor BIM, indicating that BIM may be repressed downstream of MYC (58).

As discussed above, the translation of GSIs into the clinic has been tempered by a lack of cytotoxic antitumor responses and by severe GI toxicity associated with inhibition of Notch signaling in the intestinal epithelium (24, 25). A recent study sheds light on one potential mechanism of resistance to GSI in T-ALL that might be targeted with BET bromodomain inhibitors. The authors identified a subpopulation of drug-tolerant T-ALL cells called “persisters” that developed with long-term *in vitro* GSI treatment. Because the “persister” phenotype was reversible after γ-secretase withdrawal the authors hypothesized an epigenetic mechanism of drug resistance. To test this hypothesis they performed a shRNA screen targeting nearly 350 chromatin regulator genes. Among top hits, which preferentially impaired the viability of “persister” cells while sparing the naïve population, they identified *BRD4*. Consistent with this model, “persister” cells were more sensitive to BRD4 inhibition *in vitro*, and combination therapy with GSI and JQ1 in a GSI “naïve” T-ALL xenotransplant model was more effective than either drug alone (59).

The effects of BRD inhibition are not limited to MYC transcription. For example, genome-wide expression profiling following JQ1 treatment identified *IL7R*, along with *MYC*, to be among the most down-regulated genes, and early studies correlated BRD4 inhibition with decreased IL7R expression and loss of BRD4 loading at the IL7R promoter across different cancer subtypes (60). Activating mutations in *IL7R* were recently identified in at least 9% of pediatric T-ALL, and constitutive activation of IL7R signaling in CD4−/CD8− mice thymocytes induced early T-cell precursor ALL (ETP-ALL) (61).

Finally, BRD-targeted pharmacological inhibition in T-ALL is being tested in the clinic. Currently, OTX015, a synthetic small molecule against BRD2, 3, and 4 is under evaluation in a phase 1b dose-escalation trial in relapsed or refractory AML/ALL (NCT01713582) following promising *in vivo* data showing cell-cycle arrest and apoptosis at submicromolar doses in a panel of leukemia cells (62).

Taken together, these data provide multiple lines of evidence to support testing BET bromodomain inhibitors in patients with T-ALL. Multiple BET bromodomain inhibitors have entered the clinic and are currently in testing in phase I clinical trials, including trials testing this compound class in patients with leukemia.

## D-Type Cyclins

D-type cyclins, together with cyclin-dependent kinases (CDKs), control G1 to S-phase progression and initiate DNA replication in response to mitogenic signals in many different cell types. The binding of D-type cyclins (D1, D2, and D3) to CDK4 or CDK6 leads to phosphorylation of the retinoblastoma protein (Rb). Phosphorylated Rb is inactive and unable to bind the transcription factor E2F1, leading to transactivation of E2F-dependent genes and progression to S phase. Because these proteins are widely expressed in many tissues, there was initial concern about the therapeutic window of CDK inhibitors. However, several studies have demonstrated that the absence of individual D-type cyclins is dispensable for proliferation and for the development of the majority of organs, suggesting a redundant function of these proteins in many circumstances (63–65).

Altered expression of cyclin-D genes (*CCND*) has been implicated in the pathogenesis of numerous cancers, including hematopoietic malignancies. The *t*(11;14)(q13;q32) translocation at the *CCND1* locus is a hallmark of mantle cell lymphoma (66), and molecular studies revealed activating mutations in the phosphorylation site of cyclin-D genes in DLBCL, CLL, and Burkitt lymphoma (10, 67, 68). Moreover, D-type cyclins were shown to be essential for tumor initiation *in vivo*. Mice lacking cyclin-D1 are resistant to ErbB2-driven carcinoma (69–72), while cyclin-D3-null animals are refractory to NOTCH1-induced T-ALL (73). Thus, modulation of cyclin-D CDK complexes appears a potential therapeutic strategy in several human malignancies.

Multiple groups have reported inhibition of D-type cyclins–CDK complexes as a potential therapeutic strategy for patients with T-ALL (Figure 2B). Sicinska et al. first demonstrated that cyclin-D3−/− animals are refractory to Notch1-driven T-ALL development, and knockdown of cyclin-D3 significantly inhibits proliferation in T-ALL cell lines corresponding to immature thymocytes with rearranged TCRB chains (73). Choi et al. substantiated the role of cyclin-D3 in Notch1-driven leukemia establishment *in vivo*; acute ablation of cyclin-D3 in mice transduced with activated Notch-ICD reduced the number of leukemic clones and significantly extended survival. *In vivo* pharmacological inhibition of the cyclin-D3-associated kinases CDK4/6 by PD-0332991 resulted in reduction of tumor burden and improved survival in mice transduced with activated *Notch-ICD*. In this model, treatment with PD-0332991 inhibited cell-cycle progression and induced apoptosis, phenocopying the effects of acute genetic loss of cyclin-D3. Reduced leukemic burden and prolonged survival was also seen following PD00332991 treatment in two human T-ALL cell lines xenograft models, further suggesting CDK4/6 modulation as a potential strategy for Notch-driven T-ALL malignancies (74).

A second group simultaneously reported the requirement for Cyclin-D-kinase activity in T-ALL leukemia maintenance. Sawai and colleagues reported that PD-0332991 inhibition of CDK4/6 efficiently inhibited S-phase entry in a panel of human *NOTCH1*-mutated T-ALL cell lines and in two primary T-ALL patient samples tested *in vitro*. They showed that G0/G1 arrest was associated with a decrement of pRb (S807/811), an increase in p27^Kip1^ and repression of mitosis regulator genes, such as *E2F2, CCNA2, SKP2, CDC25a, CCNE2*, and *CDT1*. Furthermore, prolonged exposure to PD-0332991 led to apoptosis as observed by a significant increase in Annexin V. Pre-clinical studies were conducted in two different *in vivo* models: an ICN1-EGFP-transduced bone marrow transplantation model in B6SJL mice and a CEM human T-ALL cell line xenograft model. PD-0332991 treatment significantly prolonged survival and dramatically decreased leukemic burden in both models. Induction of apoptosis in the ICN1-EGFP+ cells was observed in the syngeneic model. This work also demonstrated that cyclin-D3 has a unique role in the expansion of normally developing T-cell progenitors and in the induction of T-ALL. Forced *in vivo* expression of cyclin-D2 did not compensate for the lack of cyclin-D3, suggesting intrinsic differences in the function of these two proteins in T-cell development (75). Collectively, these studies demonstrate a new avenue for targeted therapies directed against cell-cycle regulators in T-ALL. With CDK4/6 inhibitors already being tested in the clinic for other diseases these findings are readily translatable.

## PI3K/PTEN/AKT/mTOR

PI3K/PTEN/AKT/mTOR is a critical pathway that elaborates both intracellular and extracellular signals to control cell metabolism, proliferation, and survival. Because several mechanisms can lead to PI3K/PTEN/AKT/mTOR activation, aberrancies of this pathway are frequently observed in human malignancies (76–79). Targeted inhibition of individual nodes in the pathway is under investigation as a cancer therapy strategy (Figure 2C). PI3K catalyzes the phosphorylation of phosphoinositide to generate the second messenger phosphatidylinositol-3,4,5 trisphosphate (PIP3) from phosphatidylinositol-3,4 bisphosphate (PIP2). PIP3 binds to the pleckstrin homology (PH) of the phosphoinositide-dependent kinase-1 (PDK-1) and the serine/threonine kinase AKT, causing both proteins to translocate to the cell membrane where they are subsequently activated. The tumor suppressor PTEN (phosphatase and tensin deleted on chromosome 10) antagonizes PI3K signaling by the dephosphorylation of PIP3. AKT can activate mTOR at least through two distinct mechanisms: directly by phosphorylation at the S2448 site or indirectly by inhibition of the tuberous sclerosis complex 2 (TSC2).

Cross talk between Notch signaling and the PI3K/PTEN/AKT pathway is under active investigation. Compelling evidence that Notch activates AKT was supported by work of Palomero and colleagues (78, 80). The authors demonstrated that HES1 (hairy and enhancer of split 1), a direct target of NOTCH1, binds to the *PTEN* promoter and represses its activity resulting in decreased PTEN expression and an increase in phosphorylation of AKT-Ser473. Thus, activated NOTCH1 signaling promotes activation of PI3K–AKT due to the transcriptional repression of PTEN. Treatment with the NOTCH1 inhibitor Compound E diminished HES1 and, as expected by the described model, restored PTEN activity. In turn, PI3K–AKT was inhibited causing impaired cellular viability. In summary, in a T-ALL background with an intact PTEN axis, GSI treatment causes a dual inhibition of Notch and PI3K–AKT signaling. Although this scenario is expected in two-thirds of T-ALL cases, deletion and/or inactivating mutations of *PTEN* have been described in 36% of primary T-ALL cases. Moreover, all told, mutations of individual components of the PI3K/AKT axis, including *PTEN*, are observed in 47% of pediatric T-ALL cases (79). In the T-ALL cases of *PTEN* deletion, despite NOTCH1–HES1 inhibition, the PI3K–AKT signaling will be maintained in an active tone, impairing full response to NOTCH1 inhibition, thus accounting for one potential intrinsic resistance mechanism to GSI treatment (80).

Inhibition of the isoforms PI3K-γ and PI3K-δ is one candidate strategy to target *PTEN* null T-ALL. Recent studies demonstrated that PI3K-γ and PI3K-δ are required for the establishment of a PTEN deficient leukemia *in vivo* (81). To test whether PI3K-γ and PI3K-δ are required for tumor maintenance, the authors developed CAL-130, a small molecule that inhibits both the catalytic domains of p110γ and p110δ. In *Pten* null mice with established T-ALL leukemia CAL-130 increased survival and reduced tumor burden in the cohort of treated animals. Furthermore, testing in T-ALL cell lines demonstrated the ability of CAL-130 to inhibit cellular growth while inducing apoptosis. Finally, cell lethality was further demonstrated in a panel of primary T-ALL samples lacking PTEN expression (81).

Because constitutive activation of PI3K signaling is common in T-ALL, PI3K inhibitors are being studied as a treatment strategy for this disease. For example, the novel dual PI3K/mTOR inhibitor NVP-BEZ235, an orally bioavailable imidazoquinoline derivative, proved to have anti-proliferative effects in several T-ALL cell lines where pro-apoptotic effects were demonstrated (82). An ongoing trial is currently testing this molecule in AML, ALL, and CML. A second example evaluated the activity of NVP-BKM120, a potent, orally available, pan-PI3K inhibitor. In human T-ALL cell lines, NVP-BKM120 induced apoptosis and cell-cycle arrest (G2/M transition). Additionally, cell lethality was confirmed in primary T-ALL lymphoblasts. As expected, NVP-BKM120 inhibited AKT as shown by a dose-dependent reduction of phosphorylation at Ser473 and at Ser235/236 and the downstream target RPS6. Finally, pre-clinical studies in a T-ALL cell line-derived xenotransplant model demonstrated that NVP-BKM120 significantly delayed tumor growth and prolonged survival (83). To date, NVP-BKM120 has been tested in a dose-escalation trial in patients with advanced solid tumors. It was safe and well tolerated and was demonstrated to inhibit the target (84). An ongoing phase I study (NCT01396499) is testing NVP-BKM120 in patients with advanced leukemia. Additionally, other studies have supported the inhibition of PI3K/AKT signaling as a treatment strategy, including through the activation of PP2A (85).

Another approach to target the PI3K pathway would be to target mTOR, the catalytic subunit of two distinct complexes mTORC1 and mTORC2. mTORC1 controls the translational regulators S6K1 and 4E-BP1, whereas mTORC2 phosphorylates AKT at Ser473 (86–88). The combined inhibition of mTOR and Notch was shown to suppress T-ALL growth (89), and several pre-clinical studies suggested that mTOR modulation can effectively reverse glucocorticoid resistance in T-ALL (90–93). Single agent mTORC1 inhibitors are not likely to be an effective therapeutic therapy as they provoke a cytostatic response and activate feedback loops to enhance cell survival (94–97). The studies described above, however, do support the testing of mTORC1 inhibitors in combinations with other drugs, and these clinical trials are now ongoing at several centers. Taken together, these studies support the continued testing of PI3K pathway inhibition as a therapeutic strategy for T-ALL.

## Conclusion

Despite unprecedented efforts to uncover the molecular complexity of T-ALL, upfront treatment is still based on cytotoxic chemotherapeutic regimens, and prognosis for this disease generally remains poor in adults and in both children and adults with relapsed disease. The paucity of effective treatment options available for patients of these unfavorable subgroups highlights the importance of improving current therapies with molecularly informed approaches. Targeting Notch, MYC/BRD4, Cyclin D3:CDK4/6, and the PI3K pathway are promising therapeutic targets in T-ALL, and the strong pre-clinical studies discussed above support further investigation of these drugs in clinical trials in patients with T-ALL.

## Conflict of Interest Statement

The authors declare that the research was conducted in the absence of any commercial or financial relationships that could be construed as a potential conflict of interest.
